# The Classification of Common Macular Diseases Using Deep Learning on Optical Coherence Tomography Images with and without Prior Automated Segmentation

**DOI:** 10.3390/diagnostics13020189

**Published:** 2023-01-04

**Authors:** Natsuda Kaothanthong, Jirawut Limwattanayingyong, Sukhum Silpa-archa, Mongkol Tadarati, Atchara Amphornphruet, Panisa Singhanetr, Pawas Lalitwongsa, Pantid Chantangphol, Anyarak Amornpetchsathaporn, Methaphon Chainakul, Paisan Ruamviboonsuk

**Affiliations:** 1 Sirindhorn International Institute of Technology, Thammasat University, Pathumthani 12000, Thailand; 2 Department of Ophthalmology, Rajavithi Hospital, Bangkok 10400, Thailand; 3 Department of Ophthalmology, Mettapracharak Hospital, Nakhon Pathom 73210, Thailand

**Keywords:** OCT, macular disease, image classification

## Abstract

We compared the performance of deep learning (DL) in the classification of optical coherence tomography (OCT) images of macular diseases between automated classification alone and in combination with automated segmentation. OCT images were collected from patients with neovascular age-related macular degeneration, polypoidal choroidal vasculopathy, diabetic macular edema, retinal vein occlusion, cystoid macular edema in Irvine-Gass syndrome, and other macular diseases, along with the normal fellow eyes. A total of 14,327 OCT images were used to train DL models. Three experiments were conducted: classification alone (CA), use of automated segmentation of the OCT images by RelayNet, and the graph-cut technique before the classification (combination method 1 (CM1) and 2 (CM2), respectively). For validation of classification of the macular diseases, the sensitivity, specificity, and accuracy of CA were found at 62.55%, 95.16%, and 93.14%, respectively, whereas the sensitivity, specificity, and accuracy of CM1 were found at 72.90%, 96.20%, and 93.92%, respectively, and of CM2 at 71.36%, 96.42%, and 94.80%, respectively. The accuracy of CM2 was statistically higher than that of CA (*p* = 0.05878). All three methods achieved AUC at 97%. Applying DL for segmentation of OCT images prior to classification of the images by another DL model may improve the performance of the classification.

## 1. Introduction

Optical coherence tomography (OCT) is a noninvasive imaging technique which provides high-resolution, cross-sectional images of macula, optic nerve head, or anterior segment structures in an eye. This device relies on the principle of light interference using the reflection of low-coherence light projected on the retina and other eye structures to construct images [1,2]. OCT images have been widely used in retina clinics worldwide to assist in diagnosis, monitoring, and treatment of macular diseases. They were also used in many pivotal clinical trials for macular diseases, such as age-related macular degeneration (AMD) [3], diabetic macular edema (DME) [4], and retinal vein occlusion (RVO) [5], to measure biomarkers at the macula as the outcomes of the trials.

Deep learning (DL) is a subset of machine learning in artificial intelligence (AI), which allows automated feature extraction [6]. Composed of multiple layers of artificial neural networks, DL led to a breakthrough in processing various important tasks in medicine, such as automated classification of tuberculosis on chest radiographs [7] or automated interpretation of echocardiography [8], with accuracies on par with medical experts in the field.

In ophthalmology, DL has been used for classification of color retinal photographs to detect referrable diabetic retinopathy (DR) with robust performance [9]. For OCT images of macula, DL has been successfully used for classification between normal and AMD [10], and for classification of patterns in OCT images of DME, such as diffuse retinal thickening, macular edema, and serous retinal detachment [11]. In another study using a big dataset of 162,721 OCT images from multiple eye centers in 5 countries, the DL software, called Pegasus-OCT, could distinguish between normal and abnormal OCT images with areas under the receiver operating characteristic curves (AUC) over 98%. Between AMD and DME, the minimum AUC values were 99% and 98%, respectively. The performance of this DL software was generally lower when the analysis was on images with insufficient quality [12]. In the classification of macular OCT images of more than two subtypes, typically of four common macular diseases (Drusen, neovascular AMD, DME, and normal), many studies achieved an accuracy, a precision, and a recall of more than 95% [13,14,15].

Image segmentation is used to separate an image into small regions, such as foreground and background. It is a key task in computer vision and has been utilized in scene understanding, image analysis, and object recognition [16]. The segmentation method can be separated into two main approaches: a region-based segmentation and a boundary-based segmentation. The region-based method considers the similarity of pixels’ features, such as intensity or texture of connected pixels, as one region. The boundary-based method finds the discontinuity of surrounding pixels to define edges.

DL was also used to perform automated segmentation of retinal layers and biomarkers on OCT images [17,18,19]. A study by Maloca et al. showed that the average intersection over union (IOU) scores for compartmentalization by DL of the vitreous, retina, choroid, and sclera were 0.9929, 0.9890, 0.8817, and 0.9768, respectively, when compared to the images compartmentalized by retinal specialists [17]. Particular techniques of automated segmentation by DL, such as active contour segmentation [18] or graph-cut theory [19], were utilized in these studies, with acceptable accuracy. Whereas automated classification of macular OCT images may be useful in the early detection of diseases, automated segmentation of the images may be useful for disease monitoring to decrease the burden of retinal specialists in busy clinics.

In a study by De Fauw et al., the authors aimed to classify macular OCT images into different diseases for providing referral recommendations of sight-threatening conditions to patients, accordingly. Using more than 15,000 macular OCT scans, the authors performed automated segmentation trained on 877 scans first, then performed automated classification trained on 14,884 of the pre-segmented macular OCT scans [20]. They could achieve robust performances on classifying eight common macular diseases, including normal OCT scans. Since image segmentation is a key task in computer vision and there were studies on DL for classification of OCT images without performing automated segmentation, a research question is posed on whether automated segmentation of the macular OCT images prior to automated classification would improve the performance, compared with using DL to perform automated classification alone without prior automated segmentation. In addition, the results of a region-based and a boundary-based segmentation are compared to demonstrate an appropriate method for preprocessing macular OCT images. We therefore conducted this study to address this question.

## 2. Materials and Methods

This study was approved by the Ethics Committee, Rajavithi Hospital, which is organized and operates according to the Declaration of Helsinki, The Belmont Report, the Council for International Organizations of Medical Sciences (CIOMS) Guidelines, and the International Conference on Harmonization of Technical Requirements for Registration of Pharmaceuticals for Human Use—Good Clinical Practice (ICH-GCP) (Protocol number is 46170 and date of approval is 5 July 2021).

**Figure 1:**
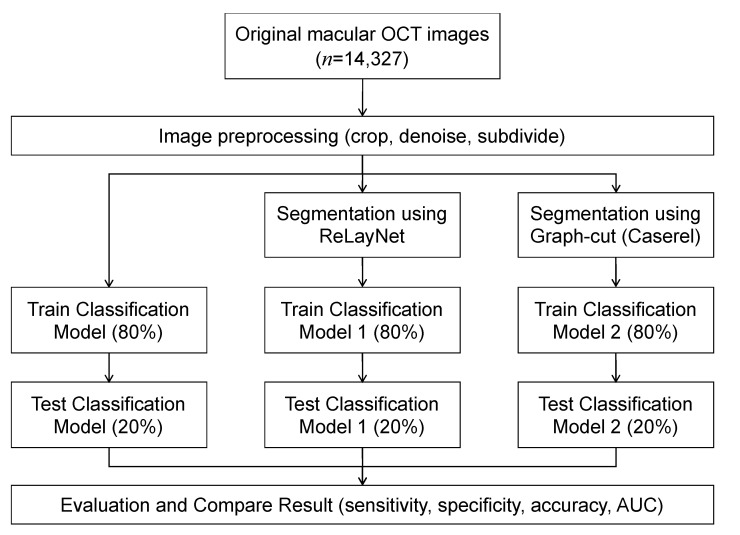
Workflow diagram.

An overview of the models is depicted in Figure 1. Each OCT image was preprocessed to improve the images’ quality by reducing noise and cropping only the macular area. The images were used as input for training the three models, where each utilized a different image segmentation method. The results of the three models were compared to evaluate the effect of the image segmentation on the classification’s performance.

**Figure 2:**
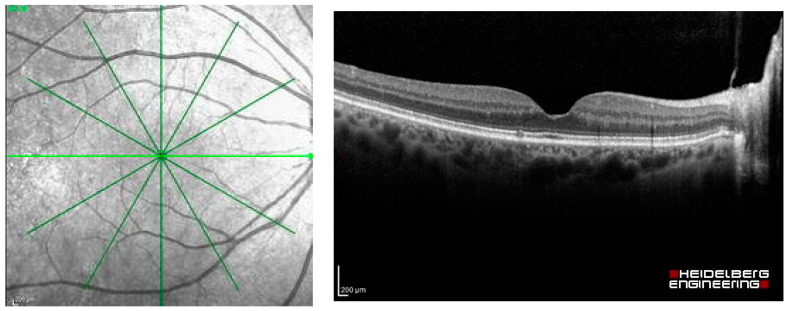
Radial OCT scan of a retina and example of an OCT image of the light green line.

The dataset used in this study was extracted as OCT images from Heidelberg Spectralis (Heidelberg Engineering, Berlin/Heidelberg, Germany), including the images of patients with common macular diseases in one eye from 1 January 2015 to 31 December 2020. The OCT images contain the radial scans from 6 lines per eye, as shown in Figure 2. They were retrieved from the visits before the patients received an intravitreal anti-VEGF injection. The OCT images from the normal fellow eyes were also retrieved in the dataset. The common macular diseases diagnosed to the eyes in the dataset were neovascular AMD (nAMD), polypoidal choroidal vasculopathy (PCV), DME, retinal vein occlusion (RVO), cystoid macular edema (CME) from Irvine-Gass syndrome, and other macular diseases that received intravitreal anti-VEGF injections at the Eye Clinic, Rajavithi Hospital. The function “Others” in the dataset refers to other relatively uncommon macular diseases, such as Stargardt’s disease, which is a hereditary disease. The appearances of the macula of these diseases are different from those of common diseases, such as PCV, DME, or RVO. We obtained a total of 14,327 OCT images, and a scan was counted as an image, as shown in Table 1.

**Figure 3:**
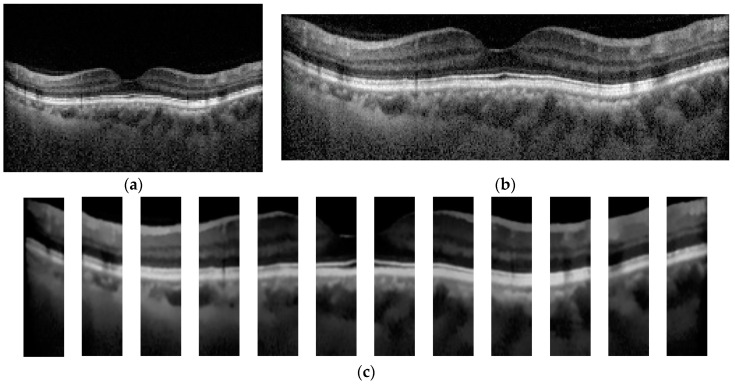
Example of original and preprocessed OCT image. (**a**) Original OCT, (**b**) Cropped OCT image, and (**c**) Denoised and subdivided OCT image.

In the step of OCT image preprocessing and image segmentation, each OCT image was cropped to a rectangular box that contains the region of the macula as much as possible, as depicted in Figure 3b. To demonstrate the effects of the image segmentation on the classification result, a mean filter was applied to each OCT image to remove noise. To improve the performance of the image segmentation in terms of the speed and quality of the segmented result, the denoised OCT image was subdivided into 12 small images (see Figure 3c). Each small image was used by the segmentation algorithm to separate the macular layers, where the size was decided from our preliminary study.

**Figure 4:**
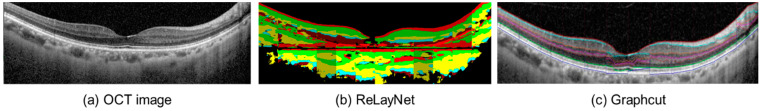
Example of the segmented OCT image (**a**) using RelayNet (**b**) and the graph-cut method (**c**).

After the modification, the images were introduced to the segmentation process. This study applied two open-source automated segmentation methods: (1) a deep convolutional neural network-based segmentation, called RelayNet [21], and (2) a boundary segmentation using the graph-cut technique [19]. The open-source RelayNet provides a set of ground-truth images that allows model training without preparing our own labels. On the other hand, the graph-cut method does not require any model training and ground truth. A comparison of the segmented results can be found in Figure 4.

RelayNet [21] is a region-based segmentation method that applies a deep convolutional neural network to assign each pixel to a particular label. It applies an encoder and a decoder to perform segmentation. The encoder is responsible for extracting important features from an input image. A contracting path of convolutional blocks, in which the size of the blocks in each layer was reduced, was employed to learn the hierarchy of contextual features and the preserved relation of the neighboring pixels. In this way, the decoder locates pixels of similar features extracted from the same region. With the contraction path of convolution blocks, the obtained segmented region was smooth due to the availability of spatial information. Lastly, each segmented region was classified by a layer classification model to assign the type, such as fluid pool and coloring the whole region, see Figure 4b for illustration.

Graph-cut [19] is an optimization image segmentation that finds the boundary among the regions in the image using the max-flow min-cut theorem [22]. It has been utilized in cardiac MR images [23]. Unlike RelayNet, graph-cut does not require prior knowledge, which are labels of each macular layer in each OCT image. Graph-cut finds the boundary of the regions using pixels’ intensity by computing a max-flow and a min-cut. The max-flow obtains when the connected pixels have a similar intensity level. These connected pixels are considered as one region. On the other hand, the min-cut obtains when the intensity of adjacent pixels is different. In other words, the flow of values between the pixels is discontinued, which is also called a cut. By discovering the cut, the boundary between the regions is found, as shown in Figure 4c. Caserel [24] is a computer-aided graph-cut segmentation of macular layers in OCT images that we used in this study as the second segmentation method.

Since a high-resolution OCT image was employed in this work, each small-preprocessed image of Figure 3c was used as an input to the two image segmentation algorithms. The segmented result of each small image was merged to acquire the image of the whole retina layer, as demonstrated in Figure 4b,c. The denoised OCT image without segmentation (classification alone method) and the segmented images from RelayNet (combined method 1) and graph-cut (combined method 2) were used as input for the OCT image classification model.

We applied a DL architecture called ResNet50 [25], implemented in Fastai [26], to create a multi-class classification model for assigning labels. An adaptive learning rate was employed for adjusting weights of the deep neural network during model training. Considering a training set of segmented images, each image was previously assigned using seven classes (class 0 = nAMD, 1 = PCV, 2 = DME, 3 = RVO, 4 = CME, 5 = Normal, and 6 = Others).

The 50-layered ResNet architecture (ResNet50) was applied with the pre-training model to assign an initial weight. For each training iteration, the images were trained with a batch size of 32, 50 epochs (iteration), adaptive learning, and the best learning rate, which was found using the cyclical learning method with stochastic gradient descent with restarts. K-fold cross-validation, where K = 5, was adopted in the experiment for training the model and determined appropriate values for each parameter, such as the learning rate. The training set was separated into five folds, where the distribution of each disease was equally distributed. The model was conducted using 80% of the obtained images for training and the remaining 20% for testing. For each fold, all six OCT images taken from each eye were assigned to be in the same fold. The experiment using each setting was conducted in five iterations, where a different fold was employed as a test for each iteration. The experiments were conducted separately to evaluate the three separate models for each image preprocessing and image segmentation method, which were denoised OCT images, segmented images using RelayNet, and segmented images using the graph-cut technique.

Sensitivity, specificity, F1-score, and accuracy were used as measurements of model performance. A 95% confident interval (CI) of the accuracy was also applied to evaluate the performance of each class. To validate the performance of the proposed models to classify OCT images, a receiver operating characteristic (ROC) curve and an area under the curve (AUC) were also depicted.

## 3. Results

After the model training, we achieved an average sensitivity of 62.55%, specificity of 95.16%, and accuracy of 93.14%, with 95% CI (92.99, 93.30), for classification of the seven classes of macula conditions in the validation using the classification alone model. The model trained using the combined method 1 with segmented OCT images processed from RelayNet as the inputs achieved an average sensitivity of 72.90%, specificity of 96.20%, and accuracy of 93.92%, with 95% CI (93.78, 94.07), which were generally higher than the model using classification alone. The model trained using the combined method 2 with segmented OCT images processed from the graph-cut technique as inputs achieved an average sensitivity, specificity, and accuracy of 71.36%, 96.42%, and 94.80%, respectively, which were generally higher than the classification alone model.

The average accuracy of the combined method 2 was higher than both the classification alone and the combined method 1. When compared between the average accuracy of the combined method 2 and the classification alone group using the dependent t-test, the difference in the accuracy was statistically significant at *p*-value = 0.0488 (*p*-value < 0.05). The F1-scores were found to follow similar trends as the accuracies for the classification of each of the conditions. Similar trends were found in the classification of each of the major macular conditions: AMD, PCV, DME, RVO, and Others, when compared among the three models.

**Figure 5:**
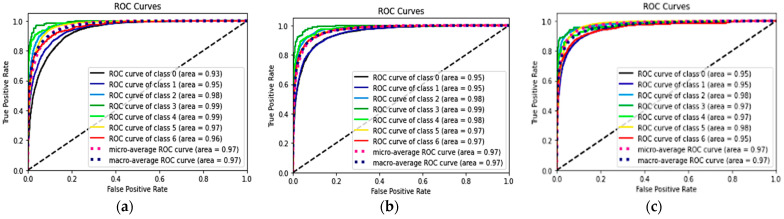
ROC and AUC of the three models: classification alone (**a**), combined method 1 (RelayNet) (**b**), and combined method 2 (graph-cut) (**c**).

Table 2 shows the performances of seven classes using the three DL models and the *p*-values of the combined methods 1 and 2, compared with the classification alone method. The ROC curves and AUC of each ROC curve of the three models for the classification of each of the seven classes showed a similar AUC at 97%. The ROC curves of each class are shown in Figure 5.

## 4. Discussion

We found in this study that the performance of the DL model for classification of common macular diseases from OCT images may be better when the OCT images are segmented before the classification. It was shown that the boundary-based image segmentation algorithm, i.e., the graph-cut technique, had better performance than the region-based method when used in combination with the automated classification. Moreover, this could be achieved without assigning a label to each segmented region on OCT images. It is possible that the segmented images of the graph-cut technique enable the DL to easily learn the texture information of each layer on the macular due to a clearly defined boundary. Compared to the results from RelayNet, each layer was replaced by a color, and the details of the macular were discarded.

Currently, the use of OCT for the diagnosis and treatment of macular diseases is ubiquitous, not only in retina clinics but in general eye clinics and in major clinical trials. With multiple scans, from 6 in routine clinical use to 128 in research, per eye, and advancements in computation power of computer hardware, an abundance of OCT images is available for research in AI. The better performance for AI to perform classification of OCT images should be helpful for identification of referrals when the OCT devices are available in primary care settings without retinal specialists. For detecting diseases in clinical care, high sensitivity is generally preferable to detect more cases. Although the AUC of the classification alone method in this study was as high as the methods with prior image segmentation, the sensitivity of the classification alone method is generally lower than the latter. From the results in Table 2, the classification of five out the six disease classes, particularly the common diseases: nAMD, PCV, DME, and RVO, had much higher sensitivity than the classification alone method. Therefore, we suggest automated segmentation prior to automated classification if DL is applied to classify OCT images for screening purposes.

The performances of classification of macular diseases using DL for the classification task alone in this study seemed to be worse than the performances reported in other studies on DL for classification of macular diseases. However, majority of these studies performed the classification for only two classes, whereas the classification in this study was for seven classes. The study by De Fauw et al. [20], in which the authors also aimed to classify macular diseases in up to eight classes, used two DL algorithms for referral recommendations: the first algorithm was for segmentation whereas the second was for classification of the segmented maps from the first algorithm. They applied an ensemble model of both segmentation and classification tasks. Five segmentation models using three-dimensional OCT images were for ensemble and the other five models were used for classification. The result of the model had an accuracy of 94.5%. Compared to the two best retina specialists, they achieved 93.3% and 93.2% accuracy. The model accuracy of each of the four referral classes, which are urgent, semi-urgent, routine, and observation, were 96.4%, 98.7%, 95.4%, and 98.4%, respectively. The semi-urgent class achieved the highest sensitivity and specificity, of 97.3% and 99.2%. Though the outcome of the previous work cannot directly compare to this work, the performance of the classification with the segmented images has confirmed the necessity of the application.

A possible explanation for the relatively low sensitivity from the classification model is this study when compared to the specificity and accuracy of the previous study, such as the study from De Fauw et al. [20], may be the use of six-line scans of OCT images for each eye. The use of six-line scans may cause a bias of having some OCT scans which may appear normal labeled as having a disease, since we labeled all the scans of the same eye as having the same disease. It is common that some scans of OCT from the diseased eye may appear normal since the scan may not pass through the lesion area. This image capture format may not provide enough data for DL to be trained. However, the objective of this study was not primarily to validate the performance of the classification model but rather to compare the performance between the classification alone and the classification in combination with segmentation models.

There are three strengths of this study. The role of image segmentation in the OCT classification was demonstrated in this study. Both region-based and boundary-based segmentation methods can improve the OCT classification performance, especially the sensitivity. Providing ground-truth images for training a DL model is known to be a labor-intensive task. Additionally, a precise ground truth affects the segmentation model’s performance. In this way, experienced ophthalmologists are needed. To cope with these problems, this work employed a graph-based image segmentation method that does not require any ground-truth and training processes. Considering an input image, it applies a min-cut and max-flow algorithm with the pixels’ value to automatically find the edge between the two regions. Lastly, utilizing six-line scan, OCT produced an acceptable result. We believe that it can be improved by assigning labels to each scan.

The limitation of this study, besides the six-line scan, is the imbalanced dataset among the number of patients with certain diseases. There were some diseases, such as CME from Irvine-Gass syndrome, that had a much smaller number of patients and OCT images. We did not use an augmentation process in this study since the number of images in each class had a high degree of difference. More data, and more balance of the data, might improve the diagnostic metrics, such as the sensitivity, specificity, and accuracy of the DL model, in future studies. The OCT device in this study was only from one brand. The applicability of the DL models to OCT images from other devices is not known. The lack of validation of our models in external datasets may be another limitation.

## 5. Conclusions

This study demonstrated that to perform automated classification of diseases on OCT images, a DL algorithm to perform automated segmentation of the images and then input the segmented images into another DL algorithm for classification of the diseases may be required to improve the performance of the classification task, even if additional computation time is a tradeoff. Future studies on the comparison of the DL models with and without automated segmentation for classification of macular diseases in new external datasets may also be required to support the findings in this study.

## Figures and Tables

**Table 1:** The distribution of OCT images assigned to each class.

Classes	Number of Images
nAMD	3761
PCV	2729
DME	1455
RVO	175
CME	192
Normal	4901
Others	1114

**Table 2:** Performance of the three models using the testing set: classification alone, combined method 1, and combined method 2 in the classification of the macular conditions.

Class	Classification Alone					Combined Method 1 (RelayNet)					Combined Method 2 (Graph-Cut)				
	Sen. (%)	Spec. (%)	F1 (%)	Acc. (%)	95% CI	Sen. (%)	Spec. (%)	F1 (%)	Acc. (%)	95% CI	Sen. (%)	Spec. (%)	F1 (%)	Acc. (%)	95% CI
nAMD	71.89	92.09	74.08	86.79	[86.24, 87.35]	70.75	95.78	77.50	89.21	[88.71, 89.72]	78.25	95.16	81.58	90.72	[90.25, 91.20]
PCV	55.73	98.15	68.14	90.07	[89.59, 90.56]	65.99	96.86	73.60	90.98	[90.51, 91.45]	70.75	97.00	77.11	92.00	[91.56, 92.45]
DME	62.88	99.11	73.67	95.43	[95.09, 95.78]	83.09	96.90	78.96	95.50	[95.17, 95.84]	76.15	98.87	81.80	96.56	[96.26, 96.86]
RVO	32.57	100.0	49.13	99.17	[99.03, 99.32]	71.42	99.84	77.63	99.49	[99.15, 99.43]	60.57	99.92	72.35	99.43	[99.31, 99.56]
CME	45.83	99.96	61.75	99.23	[99.33, 99.57]	39.06	99.96	55.14	99.14	[99.00, 99.30]	45.83	99.90	59.86	99.18	[99.03, 99.32]
Normal	98.28	79.03	82.38	85.62	[85.05, 86.20]	89.90	92.26	87.80	91.45	[91.00, 91.91]	96.95	86.10	86.69	89.82	[89.32, 90.31]
Others	70.64	97.77	71.70	95.66	[95.33, 96.00]	90.12	91.80	62.73	91.67	[91.22, 92.13]	71.00	97.98	72.83	95.88	[95.56, 96.21]
Average	62.55	95.16	68.69	93.14	[92.99, 93.30]	72.90	96.20	73.34	93.92	[93.78, 94.07]	71.36	96.42	76.03	94.80	[94.66, 94.94]
*p*-Value									0.5106					0.0488	

## Data Availability

The data are partially available at https://2021.asiateleophth.org/big-data-competition/ (1 December 2021).
